# Optimal Biofunctionalization of Gold Nanoislands for Electrochemical Detection of Soluble Programmed Death Ligand 1

**DOI:** 10.1002/smsc.202400411

**Published:** 2024-09-26

**Authors:** Zahra Lotfibakalani, Borui Liu, Monalisha Ghosh Dastidar, Thành Trân‐Phú, Krishnan Murugappan, Parisa Moazzam, David R Nisbet, Antonio Tricoli

**Affiliations:** ^1^ Nanotechnology Research Laboratory Faculty of Engineering The University of Sydney Darlington NSW 2008 Australia; ^2^ Nanotechnology Research Laboratory Research School of Chemistry Australian National University Canberra ACT 2601 Australia; ^3^ Mineral Resources CSIRO Private Bag 10 Clayton South VIC 3169 Australia; ^4^ The Graeme Clark Institute The University of Melbourne Melbourne VIC 3010 Australia

**Keywords:** aptasensors, electrochemical detections, gold nanoislands, point‐of‐care biosensors, soluble programmed death ligand‐1

## Abstract

Soluble programmed death ligand‐1 (sPD‐L1), a pivotal immune checkpoint protein, serves as a biomarker for evaluating the efficacy of cancer therapies. Aptamers, as highly stable and specific recognition elements, play an essential role in emerging point‐of‐care diagnostic technologies. Yet, crucial advancements rely on engineering the intricate interaction between aptamers and sensor substrates to achieve specificity and signal enhancement. Here, a comprehensive physicochemical characterization and performance optimization of a sPD‐L1 aptamer‐based biosensor by a complementary set of state‐of‐the‐art methodologies is presented, including atomic force microscopy‐based infrared spectroscopy and high‐resolution transmission electron microscopy, providing critical insights on the surface coverage and binding mechanism. The optimal nanoaptasensors detect sPD‐L1 across a wide concentration range (from am to μm) with a detection limit of 0.76 am in both buffer and mouse serum samples. These findings, demonstrating superior selectivity, reproducibility, and stability, pave the way for engineering miniaturized point‐of‐care and portable biosensors for cancer diagnostics.

## Graphical abstract

Soluble programmed death ligand‐1 (sPD‐L1) is an important immune checkpoint protein used as a biomarker for cancer therapy efficacy. Aptamers, known for their stability and specificity, play an essential role in emerging point‐of‐care diagnostic technologies. This study provides a comprehensive physicochemical characterization of the sPD‐L1 aptamer‐based biosensor to optimize performance and assess surface coverage and binding mechanisms..

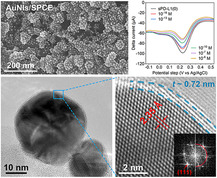

## 1 Introduction

Programmed death ligand 1 (PD‐L1), a member of the B7‐CD28 protein family, plays a pivotal role in regulating the activation and function of immune cells, particularly T cells. This protein is notably overexpressed in various cancers, making its detection crucial for both diagnostics and treatment strategies.^[^ ^1^ ^]^ When PD‐L1 binds to its receptor, programmed cell death protein‐1 (PD‐1), it suppresses T‐cell activity, enabling cancer cells to evade the immune system and promote tumor growth.^[^ ^2^, ^3^ ^]^ Blocking the PD‐L1/PD‐1 pathway has demonstrated potential in enhancing immune responses. Monoclonal antibodies targeting this interaction effectively enhance T cell recognition of tumors.^[^ ^4^ ^]^ Assessing PD‐L1 levels is, therefore, vital in predicting treatment responses to immune checkpoint inhibitors.

PD‐L1 can exist in multiple forms, including membrane bound, exosomal, and soluble (soluble programmed death ligand‐1 [sPD‐L1]) due to post‐translational modifications. Notably, sPD‐L1, which circulates freely in body fluids, is often found at higher levels in cancer patients than in healthy individuals and is associated with poorer prognoses.^[^ ^1^, ^2^, ^3^ ^]^ Enzyme‐linked immunosorbent assay (ELISA) is commonly used to measure sPD‐L1 levels in blood, influenced by various factors like gender, tumor stage and size, and grade. For instance, studies on nonsmall‐cell lung cancer report a large range of concentrations from 3.84 nm up to 27 pm in plasma and serum.^[^ ^4^, ^5^ ^]^ However, despite its utility, ELISA's time‐consuming nature and reliance on specialized equipment limit its broader application, particularly in resource‐limited settings, where delays in testing could pose significant threats to patient lives. This necessitates the development of alternative techniques for sPD‐L1 detection that may more easily be used in decentralized settings.

Electrochemical biosensor technology, particularly suited for point‐of‐care applications, offers a rapid and straightforward detection method. Electrochemical immunosensors using antibodies or antigens as bioreceptors have been proposed for sPD‐L1 detection.^[^ ^6^, ^7^, ^8^ ^]^ However, the use of antibodies is often hampered by issues such as size, stability, and cost. In contrast, aptamers—short, single‐stranded oligonucleotides—provide a more advantageous approach due to their chemical stability, scalability, and high‐affinity binding capabilities. The smaller size of aptamers also allows them to access protein epitopes less reachable by the larger antibodies.

Despite the stability of aptamers as biorecognition elements, crucial advancements rely on engineering the intricate interaction between aptamers and sensor substrates to achieve signal enhancement and specificity. In this study, we investigated this interaction using transmission electron microscopy (TEM) and atomic force microscopy‐based infrared spectroscopy (AFM‐IR) techniques for detecting and quantifying sPD‐L1, a significant biomarker in early‐stage cancer diagnosis and noninvasive monitoring of tumor immunotherapy. Our sensor relies on the dispersion of gold nanoislands (Au NIs) on screen‐printed carbon electrodes (SPCE), thereby enhancing both sensitivity and selectivity. Our design employs a single‐stranded DNA aptamer (ss‐DNA aptamer), covalently attached to the Au NIs through thiol chemistry, to serve as a bioreceptor specifically targeting sPD‐L1. The extensive active surface of the Au NIs, combined with the aptamer's selectivity and stability, contributes to a highly sensitive probe, significantly lowering detection limits in varied test environments, including spiked buffer and serum samples. Furthermore, our aptasensor demonstrates exceptional selectivity for sPD‐L1 even in the presence of common interfering biomolecules such as insulin, glucose, and glycine, underscoring its practical potential in the early detection and monitoring of cancer. This study allows us to quantitatively analyze, at the nanoscale, the correlation between the rational design of the sensors’ nanostructures and their outstanding capabilities, providing valuable insights that can guide future research in relevant fields.

## 2 Results and Discussion

**Figure 1:**
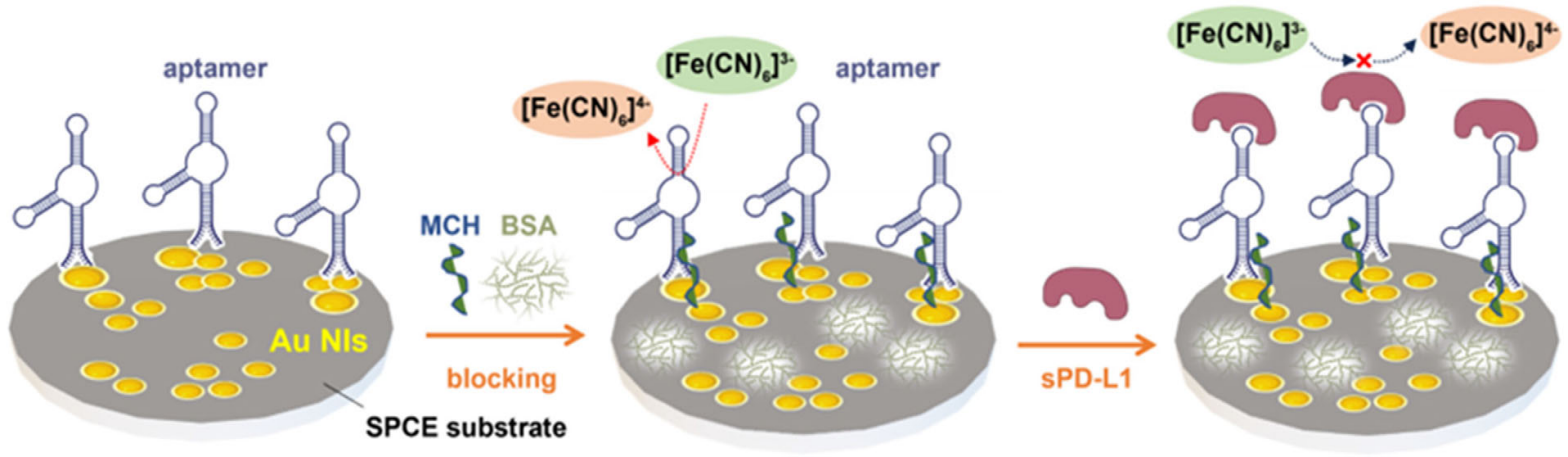
Schematic of the biosensor layout for sPD‐L1 detection: Au NIs/SPCE surface with ss‐DNA aptamer, MCH backfilling, and BSA passivation. Electron transfer is enhanced between the redox probe ([Fe(CN)_6_]^4−^/[Fe(CN)_6_]^3−^) and the SPCE substrate by the Au NIs. Target protein interaction with aptamer inhibits redox probe access to Au NIs, reducing the redox current.

**Figure** **1** illustrates the sPD‐L1 biosensor fabrication approach. A thiol‐containing DNA aptamer, which has high specificity and affinity and undergoes a large conformational change when binding to sPD‐L1,^[^ ^9^ ^]^ is used to functionalize the surface of Au NIs. The dissociation constant (*K*_d_) is a crucial indicator for assessing the affinity of aptamers. This aptamer's *K*_d_ is 51.34 nm,^[^ ^9^ ^]^ indicating strong binding. To minimize nonspecific adsorption of proteins and other impurities, 6‐mercapto‐1‐hexanol (MCH) and bovine serum albumin (BSA) are applied for the passivation of the Au NIs and carbon substrate sites not bonded to the aptamer, following methods established previously for GA^[^ ^10^ ^]^ and miRNA^[^ ^11^ ^]^ electrochemical detection. A ferricyanide redox probe was employed to derive the sPD‐L1 concentration from the residual Au sites available for the probe reaction. The binding of sPD‐L1 to the functionalized Au NIs alters the reduction and oxidation reactions of the ferricyanide probe at the working electrode, a process quantifiable through cyclic voltammetry (CV) and differential pulse voltammetry (DPV) methods.

**Figure 2:**
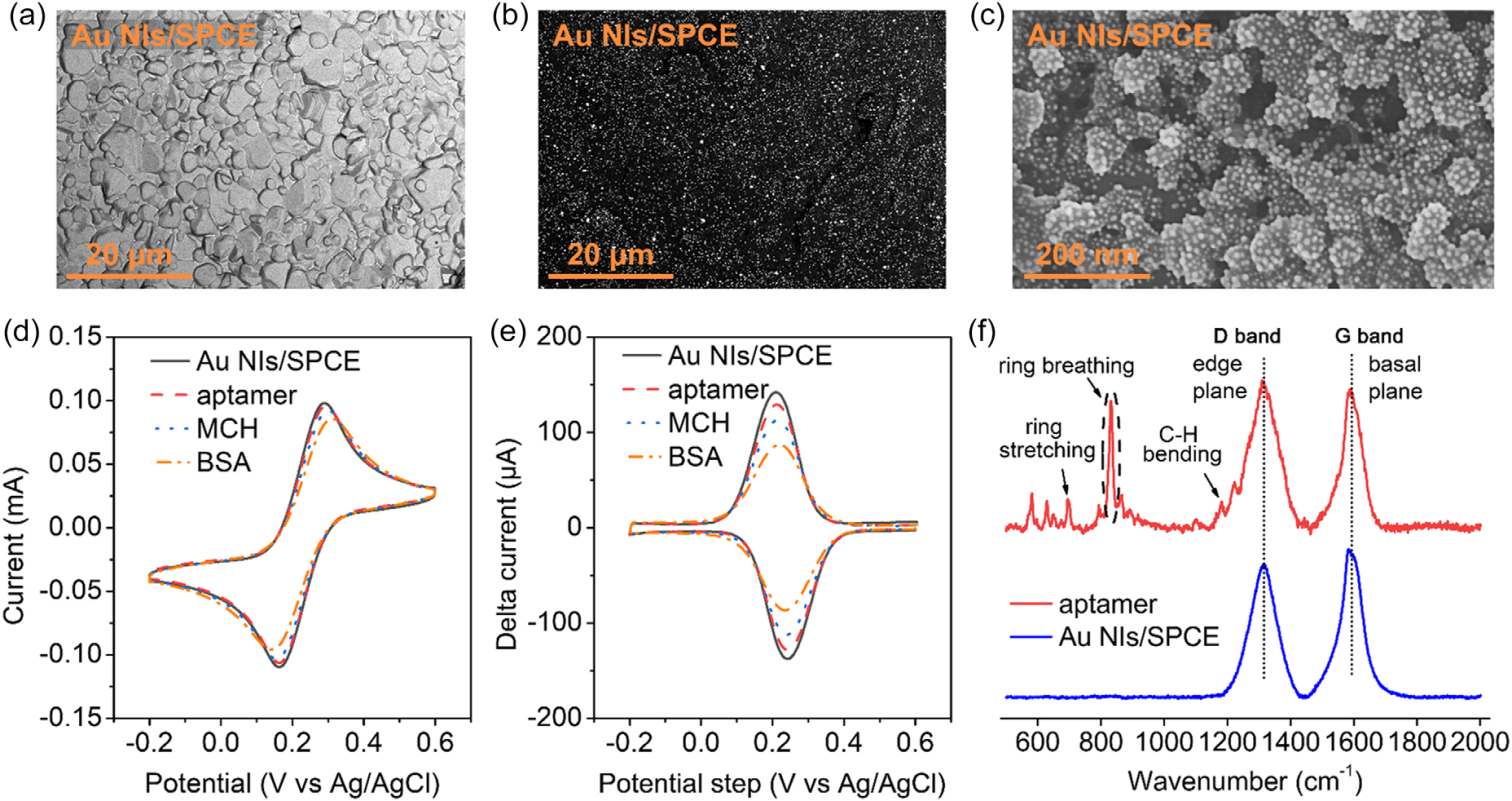
a) Microstructure of the Au NIs/SPCE electrode. b) High‐contrast SEM image of FSP‐deposited Au NIs on SPCE substrate. c) High‐resolution SEM image of Au NIs on electrode surface. Characterization of surface functionalization by d) CV and e) DPV in 0.1 m PBS solution containing 5 mm ferricyanide of a bare Au NIs/SPCE surface (black line), aptamer‐immobilized Au NIs/SPCE (red), MCH (blue), and BSA (orange)‐treated Au NIs/SPCE. f) Raman spectrum of the Au NIs/SPCE surface (black) and functionalization with the ss‐DNA aptamer (red).

**Figure 3:**
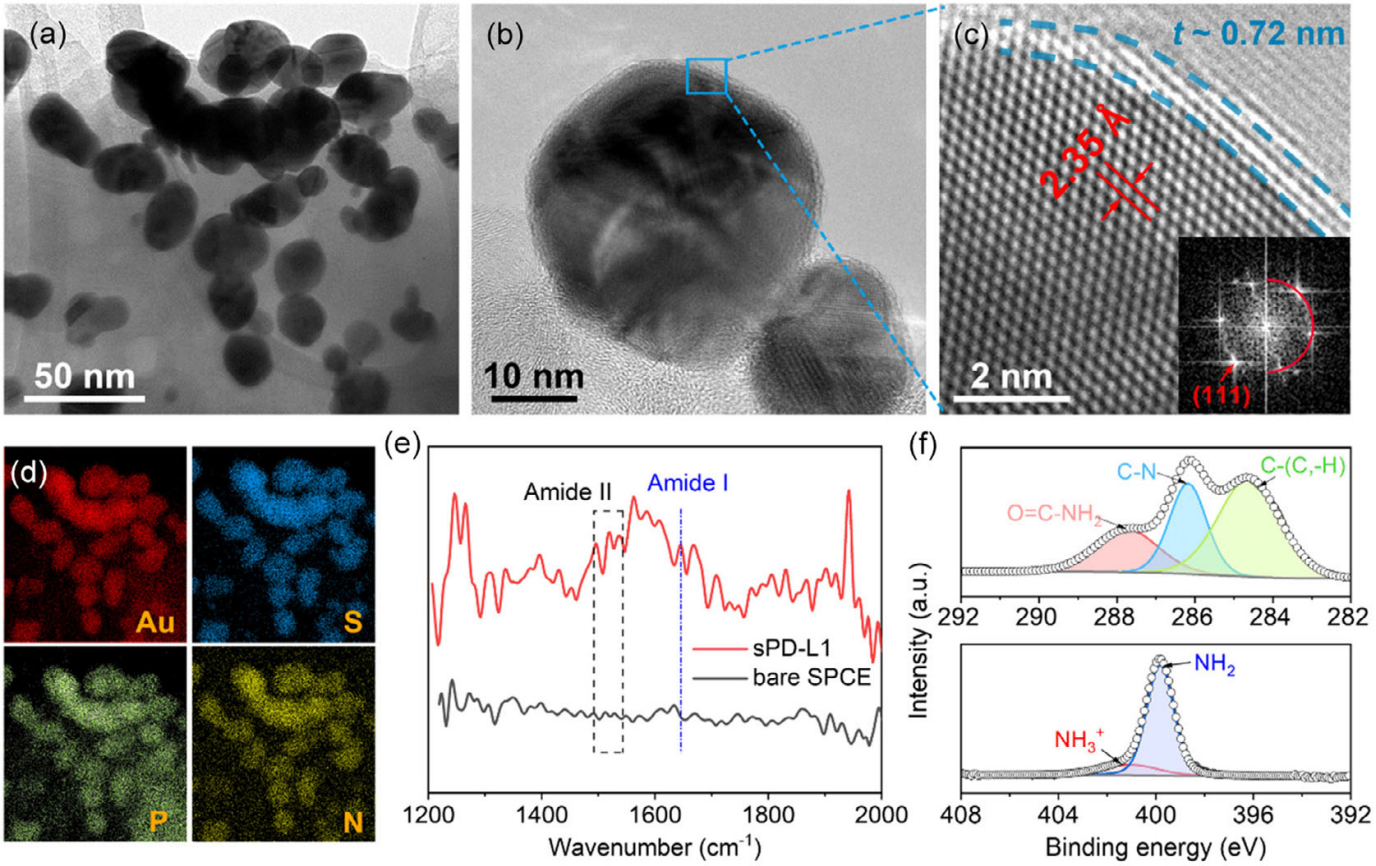
Material characterizations of the aptamer‐functionalized Au NIs upon exposure to sPD‐L1 protein. a,b) TEM images of the functionalized Au NIs upon sPD‐L1 exposure. c) High‐resolution TEM image of the functionalized Au NIs upon sPD‐L1 exposure. d) Elemental mapping of Au, S, P, and N, showing a uniform sPD‐L1 nanolayer on Au NIs. e) AFM‐IR results of the functionalized Au NIs upon sPD‐L1 exposure on SPCE and bare SPCE in the spectral range of 1200–2000 cm^−1^. f) XPS spectrum of C1s and N1s of aptasensor after binding of sPD‐L1.

The morphology and distribution of the deposited Au NIs on the SPCE substrate are shown in **Figure** **2**a–c. Figure 2a shows the scanning electron microscopy (SEM) image of the topographical morphology of the Au NIs/SPCE electrode. At this magnification, resolving individual Au NIs is challenging due to their small size (*d *≈ 19 nm, see also Figure S2, Supporting Information). However, Figure 2b,c clearly demonstrates the overall uniform distribution of Au NIs across the SPCE surface. This uniform deposition of Au NIs achieved by flame spray pyrolysis (FSP) facilitates the formation of a high density of active sites on the electrode surface, further enhancing the sensitivity and detection limit of our biosensor. Figure 2d presents the CV plots recorded at each stage of surface modification. Notably, the intensities of both the anodic and cathodic peaks of ferricyanide decrease significantly upon aptamer binding. This reduction is primarily ascribed to the electrostatic repulsion between the negatively charged redox probe and the aptamer's phosphate backbone. Additionally, the decrease in accessible surface area for redox reactions contributes to the observed reduced peak current.^[^ ^12^, ^13^ ^]^ This hindered diffusion of the redox probe to the electrode surface is caused by the physical barrier formed by the aptamer layer. Subsequent application of MCH and BSA further decreases the peak current, indicating further reduction in the surface area available to the redox probe. This suggests effective passivation of the surface not functionalized by the aptamer. In Figure 2e and S1, Supporting Information, the DPV data corroborates the CV findings, displaying a consistent decline in peak current changes. These measurements were conducted using at least three sensors prepared via the same method, as detailed in Figure 1–**3** and in the Experimental Section.

Figure 2f illustrates the Raman spectroscopy analysis of the SPCE (in black) and the aptamer‐modified Au NIs/SPCEs (in red). The Raman spectrum of the SPCE reveals D band peaks at 1348 cm^−1^ and G band peaks at 1588 cm^−1^, indicative of edge plane (disorders or defects) and basal *sp* ^2^ in‐plane vibrations in the carbon atoms.^[^ ^14^, ^15^ ^]^ While the specific Raman peaks of an aptamer can vary depending on its base pair composition, sequence, and resulting molecular conformation, the most pronounced Raman bands for both DNAs and RNAs typically fall within the 600–1700 cm^−1^ range.^[^ ^16^ ^]^ Upon functionalization of the working electrode with the DNA aptamer, a distinct peak at 830 cm^−1^ was observed, which aligns with the ring breathing characteristic of DNA aptamers,^[^ ^17^ ^]^ confirming its presence and successful attachment to the electrode surface. Moreover, various distinct vibrational modes associated with the nucleotide bases were detected. The C–H bending vibrations of these bases manifested at 1180 cm^−1^, while the stretching vibrations were evident at 690 cm^−1^. These vibrational patterns validate the successful functionalization of the working electrode with the DNA aptamer.

The binding of the Au NIs with the aptamer and subsequently with sPD‐L1 was further analyzed using TEM. TEM images (Figure 3a–c) of the electrode display a uniform functionalization layer of the sPD‐L1 protein (10 pm concentration) with an approximate thickness (*t*) of 0.72 nm on the Au NIs. TEM images of the Au NIs functionalized solely with the aptamer are not included, as the aptamer layer is too thin to be distinctly visualized. In addition, energy dispersive X‐ray spectroscopy mapping (Figure 3d) shows the distribution of elements including gold (Au), sulfur (S), phosphorus (P), and nitrogen (N) on the Au NIs. The presence of sulfur is attributed to the thiolated aptamers used for functionalization. Moreover, phosphorus and nitrogen, essential elements in nucleic acids, were detected: phosphorus in the sugar‐phosphate backbone and nitrogen in nucleobases of aptamers. These elements’ presence is indicative of the aptamers and potentially bound proteins.^[^ ^18^, ^19^ ^]^ These mapping results provide concrete evidence of the successful functionalization of the working electrode with the aptamer and the subsequent binding of sPD‐L1.

Figure 3e illustrates the AFM‐IR results of sPD‐L1‐functionalized Au NIs on an SPCE substrate. In comparison to the bare SPCEs, the AFM‐IR spectrum of the sPD‐L1 aptasensor reveals two distinct and pronounced bands (Figure 3e). The broader ‘amide I’ band at 1643 cm^−1^ is associated with the stretching vibrations of the carbonyl (C=O) functional group, an indicator of peptide bonds in proteins like sPD‐L1. Simultaneously, the “amide II” band appears within the 1500–1600 cm^−1^ range. This band results from a combination of bending vibrations related to the N—H bond observed at 1521 cm^−1^ and the characteristic C—N stretching vibrations. The emergence of these distinct and prominent bands in the AFM‐IR spectrum again confirms the successful binding of sPD‐L1 on the aptasensor.

Moreover, the X‐ray photoelectron spectroscopy (XPS) survey spectrum (Figure 3f) further corroborates the presence of the sPD‐L1 protein. The survey spectrum (Figure S3, Supporting Information) evidences the presence of C1*s* and N1*s* peaks, expected elements within the sPD‐L1 protein structure. Additionally, the deconvoluted C1s spectra exhibit three peaks at 287.8, 286.1, and 284.7 eV, respectively, corresponding to O=C—NH_2_ (amide), C—N (imide), and C—(C, H) (aromatic) bonds, aligning with the expected chemical functionalities present in sPD‐L1.^[^ ^20^ ^]^ The N1s spectra are deconvoluted into two peaks at 401 and 399.1 eV, representing nitrogen in the NH_3_^+^ (primary amine) and NH_2_ (secondary amine) groups, respectively, further confirming the presence of the protein.^[^ ^20^ ^]^ These XPS findings, in conjunction with the TEM observations, provide a comprehensive picture of the successful binding and immobilization of sPD‐L1 onto the aptasensor surface.

**Figure 4:**
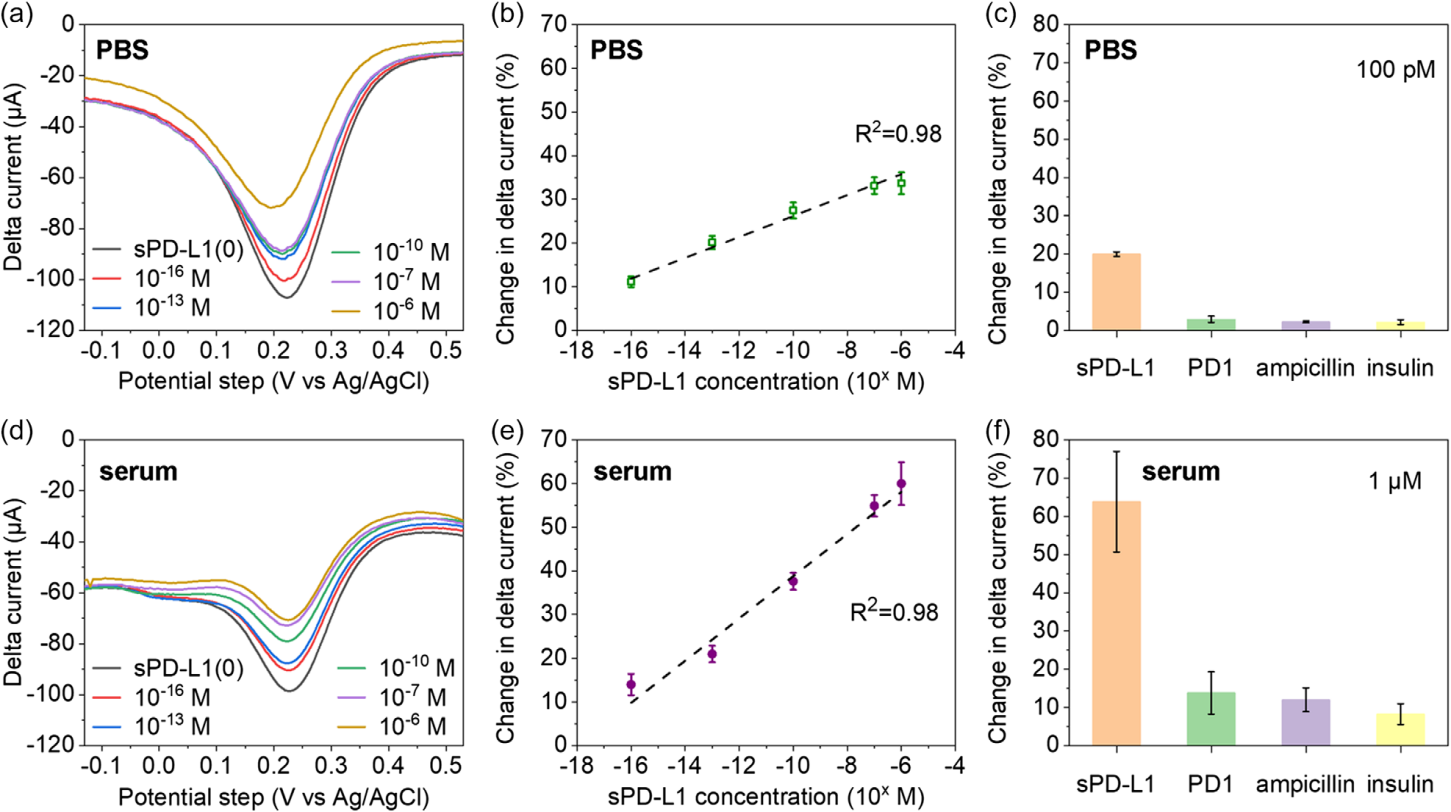
a) DPV response of aptasensor as a function of sPD‐L1 concentration from 10^−16^ to 10^−6^ m in 0.1 m PBS. b) Calibration plot for sPD‐L1 sensing in sPD‐L1 concentration range from 10^−16^ to 10^−6^ m in 0.1 m PBS. c) Selectivity for sPD‐L1 against PD1, ampicillin, and insulin in 0.1 m PBS at a concentration of 100 pm. d) DPV responses of aptasensor as a function of sPD‐L1 concentration from 10^−16^ to 10^−6^ m in mouse serum at 1:10 dilution. e) Calibration plot for sPD‐L1 sensing in sPD‐L1 concentration range from 10^−16^ to 10^−6^ m in mouse serum at 1:10 dilution. f) Selectivity for the sPD‐L1 against PD1, ampicillin, and insulin in mouse serum (1:10 dilution) at a concentration of 1 μm.

Building upon the optimization of our aptasensor binding, we further investigated its performance in detecting soluble sPD‐L1. **Figure** **4**a displays the response of the aptasensor to soluble sPD‐L1 in a phosphate‐buffered saline (PBS) solution, characterized using DPV across a concentration range from 10^−16^ to 10^−6^ m. A gradual decrease in the ferricyanide peak current was observed with increasing concentrations of sPD‐L1. The sensor's calibration curve (Figure 4b) exhibits a linear response to sPD‐L1, indicated by a high correlation coefficient (*R* ^2^ = 0.994, *S*/*N* = 3) and a low limit of detection (LOD) of 0.76 am. The consistently low standard deviations across all tested concentrations indicate a high level of reproducibility in the aptasensor preparation. This low variability confirms the reliability and consistency of sPD‐L1 sensing results. Our comparative study shows (Figure S4, Supporting Information) that our Au NIs provide superior sensor performance over the commercial Au NPs. This may be due to the high‐temperature process of FSP that fosters a more intimate contact between the Au NIs to SPCE, improving durability and stability under assay conditions. This combined with the improved electron transfer properties of the Au NIs seems to contribute a more reliable and effective performance for the sensitive and specific detection of analytes, such as 1 pm sPD‐L1, tested here. In evaluating the selectivity of the aptasensor, various proteins and biomolecules including sPD‐L1, PD1 (its cognate receptor), ampicillin, and insulin were tested on the sensor. The results (Figure 4c) showed a notably greater decrease in delta current (by a factor of five) for sPD‐L1 compared to other analytes. This exceptional specificity demonstrates the effectiveness of the aptamer in discriminating between sPD‐L1 and structurally similar molecules like PD1, a critical factor considering their potential for cross‐reactivity and interference in detection.

To assess the sensor's performance in complex media, simulating the complexity of human serum, various concentrations of sPD‐L1 were spiked into different batches of mouse serum (M5905, Sigma Aldrich) at 1:10 dilution, ranging from 10^−16^ to 10^−6^ m. The sensor demonstrated remarkable sensitivity, detecting sPD‐L1 concentrations as low as 10^−16^ m, a sensitivity level consistent with that observed in PBS solution (Figure 4a,b), and a small standard deviation of 1.2%. This small standard deviation observed during the detection of sPD‐L1 concentrations in various media indicates an excellent specificity and batch‐to‐batch reproducibility of our aptasensor as well as its stability over time (Figure S5, Supporting Information). Notably, the aptasensor exhibited a broad linear detection range from 10^−16^ to 10^−6^ m (Figure 4e), indicating its potential for diagnostic applications in scenarios involving subpicomolar sPD‐L1 concentrations. Stability, as a critical performance indicator for aptasensors, is essential for their practical application. In this study, we prepared a series of aptasensors and stored them at 4 °C to monitor their detection responses daily. As shown in Figure S5, Supporting Information, the aptasensors demonstrated a stable performance with less than 12% response decay after 14 days. The consistent sensitivity observed in detecting sPD‐L1 concentrations across various media confirms that the aptasensors maintained their performance without deterioration over time, demonstrating consistent reliability across different sensors. To validate the performance of the developed electrochemical aptasensor for sPD‐L1 detection, we used nontarget proteins like PD‐1 and insulin, as well as biomolecules like glucose and glycine, as controls. Furthermore, the sensor demonstrated superior selectivity in complex serum matrices, evidenced by a 60% delta current change for sPD‐L1 (Figure 4f), compared to lower selectivity rates for PD1, ampicillin, and insulin (i.e., 14%, 13%, and 11%, respectively). However, it is noteworthy that while maintaining high selectivity, the sensor's response in diluted mouse serum showed slightly larger standard deviations at each concentration point compared to PBS solutions which is attributed to the diverse composition of biological samples.

**Table** **1** presents a comparative analysis of the analytical performance between our nanoscale aptasensor architecture and other existing electrochemical biosensors for sPD‐L1 detection. Our aptasensor architecture shows a significant higher sensitivity and lower LOD (0.76 am) than the gold‐standard ELISA technique (Figure S6, Supporting Ifnormation), having a sPD‐L1 detection limit of 0.43 pm. The remarkable sensitivity of our electrochemical aptasensors can be attributed to its nanoarchitecture and the advantages of electrochemical detection methods, such as low background noise and high signal amplification. Besides, it can be due to the high affinity and stability of the aptamer used for sPD‐L1 targeting. While several studies have employed antibodies as biorecognition elements for sPD‐L1 detection, these antibodies often suffer from increased cross‐reactivity with nontarget analytes and decreased stability under temperature and pH fluctuations, negatively impacting sensor accuracy.^[^ ^6^, ^7^ ^]^ In contrast, aptamers are smaller and more stable, offering reduced cross‐reactivity and faster binding kinetics. This advantage of aptamers improves sensor response time and enables more precise targeting. Consequently, the adoption of aptamers as biorecognition elements significantly boosts the sensor's reliability and efficiency, paving the way for their broader application in clinical diagnostics and analysis.

**Table 1:** Comparison of recent studies on electrochemical biosensors for the detection of sPDL‐1.

Biorecognition element	Substrate	E‐chem method	LOD	Linear range	Media	Preparation [h]	Analysis [min]	References.
Aptamer	Au NIs‐SPCE	DPV	0.76 am	100 am–1 μm	serum	4	30	This work
Aptamer	Au NPs	DPV	10 pm	10 pm–2.5 nm	serum	72	30	[21]
Bistrol‐Myers Sqibb – compound 8	Au electrode	EIS	100 fm	10 am–100 nm	PBS	24	60	[22]
Antibody	Au NPs	SWV	15 am	13.8 am–0.138 nm	whole blood	3	–	[6]
Antibody	Au NPs	DPV	10 pm	10 nm–10 pm	PBS	4	–	[7]
Antibody	SPCE	amperometry	86 pm	240–5000 pm	serum	4	60	[23]
Antibody	Single‐walled CNTs	DPV	5 pm	5 pm–5 nm	serum/plasma	24	20	[24]

## 3 Conclusion

Here, we have successfully developed an electrochemical aptasensor nanoscale architecture for the detection of the sPD‐L1 protein, an important biomarker in cancer diagnostics and therapeutic efficacy monitoring, demonstrating exceptional sensitivity and selectivity. Advanced characterization techniques, such as TEM and AFM‐IR, suggest the binding of a nanothin layer of aptamer to the Au NIs, the effective passivation of these nanoparticles, and their precise recognition of the sPD‐L1 protein. The aptamer's optimized binding and the sensor surface's passivation process culminated in a biosensing platform characterized by a remarkably low detection limit of 0.76 am and a wide linear detection range from 100 am to 1 μm. The biosensor also demonstrated excellent selectivity for sPD‐L1, distinguishing it from various biomolecules such as PD1, ampicillin, and insulin in diverse environments, in both buffer solutions and diluted mouse serum samples. Importantly, our aptamer‐based approach addresses the challenges of stability and complex synthesis typically associated with conventional antibody‐based methods. These results underscore the potential of our aptasensor as a compact, highly effective tool for the rapid and sensitive point‐of‐care diagnosis and monitoring of cancer treatment.

## Conflict of Interest

The authors declare no conflict of interest.

## Author Contributions

**Zahra Lotfibakalani**: Conceptualization (lead); Data curation (lead); Formal analysis (lead); Investigation (lead); Methodology (lead); Software (lead); Validation (lead); Visualization (lead); Writing—original draft (lead); Writing—review & editing (lead). **Borui Liu**: Data curation (lead); Formal analysis (lead); Investigation (lead); Methodology (lead); Project administration (lead); Supervision (lead); Validation (lead); Visualization (lead); Writing—original draft (lead); Writing—review & editing (lead). **Monalisha Ghosh Dastidar**: Data curation (supporting); Methodology (supporting). **Thành Trân‐Phú**: Data curation (supporting); Formal analysis (supporting); Investigation (supporting): **Krishnan Murugappan**: Supervision (supporting). **Parisa Moazzam**: Conceptualization (supporting); Investigation (supporting); Methodology (supporting). **David Nisbet**: Resources (lead); Supervision (lead). **Antonio Tricoli**: Conceptualization (lead); Data curation (lead); Formal analysis (lead); Funding acquisition (lead); Methodology (lead); Project administration (lead); Resources (lead); Supervision (lead); Validation (lead); Visualization (lead); Writing—original draft (lead); Writing—review & editing (lead).

## Supporting information

Supplementary Material

## Data Availability

The data that support the findings of this study are available from the corresponding author upon reasonable request.
