# Supplementary material for: Optimal Biofunctionalization of Gold Nanoislands for Electrochemical Detection of Soluble Programmed Death Ligand 1
Source: Small Sci. 2024 Sep 26;5(1):2400411. doi: 10.1002/smsc.202400411 (PMC11935212; doi:10.1002/smsc.202400411)
Supplement: Supplementary file 1 — Supplementary Material [file SMSC-5-2400411-s001.pdf]

Supporting Information

Optimal Bio-Functionalization of Gold Nanoislands for Electrochemical Detection of Soluble Programmed Death-Ligand 1

Zahra Lotfibakalani,[a] Borui Liu,[a,*] Monalisha Ghosh Dastidar,[b] Thanh Tran-Phu,[b] Krishnan Murugappan,[b,d] Parisa Moazzam,[a,b] David Nisbet,[c] Antonio Tricoli,[a,b,*]

a. Nanotechnology Research Laboratory, Faculty of Engineering, The University of Sydney, Darlington, NSW, 2008 Australia

b. Nanotechnology Research Laboratory, Research School of Chemistry, Australian National University, Canberra, ACT, 2601 Australia

c. The Graeme Clark Institute, The University of Melbourne, Melbourne, VIC, 3010 Australia

d. CSIRO, Mineral Resources, Private Bag 10, Clayton South, VIC, 3169 Australia

* Corresponding author.

E-mail: antonio.tricoli@sydney.edu.au, borui.liu@sydney.edu.au

Experimental Methods

1. **Materials and reagents:** Potassium ferricyanide (III) ($K_3Fe(CN)_6$), tris(2-carboxyethyl) phosphine (TCEP), 6-mercapto-1-hexanol (MCH), potassium nitrate (KNO_3), Gold nanoparticles (Au NPs) and tris-EDTA (TE) buffer solution (pH=8) were obtained from Sigma-Aldrich Inc. (Australia). PBS (pH=7.2) was obtained from ThermoFisher Scientific Inc. (Australia). SPD-L1 was purchased from Acro biosystems. The DNA aptamer for sPD-L1 was acquired from Integrated DNA Technologies LCG Biosearch Technologies. It consisted of 38 bases (AAG ACG GAC CAG CCT TGC CGC AAG ACG GAC CAG GGA TT) and was chemically modified on both ends. The screen-printed carbon electrodes (SPCEs) were purchased from Metrohm DropSens, made of a ceramic substrate with a 4 mm working electrode (DRP-C110).
2. **Preparation of Au NIs/SPCEs:** Au-modified electrodes have been prepared by high-temperature flame spray pyrolysis technique as described previously which has been named with Au nanoislands.[1] Briefly, 0.01 m $HAuCl_4 \cdot 3H_2O$ was diluted in ethyl alcohol, dispensed into a fine spray, and finally deposited on the surface of screen-printed carbon electrodes. For the comparative study, Au NPs (20 nm, Sigma-Aldrich Inc.) were used to fabricate Au NPs/SPCE. The working electrode was modified with Au NPs via the drop-casting technique [2] and allowed to dry at room temperature, resulting in the Au NPs/SPCE electrode.
3. **Preparation and immobilization of DNA aptamer:** The dithiol-modified aptamer was reduced by 10 mM TCEP (1:1, v/v) at room temperature for 1 hr. After that, TE buffer (pH=8) was added to bring the stock to the final volume and incubated on Au NIs/SPCEs for 3 hours at room temperature.
4. **Biosensor fabrication:** The DNA immobilized sensor was immersed into the MCH solution (2.0 mM) for 1 hour at room temperature. Then the surface was treated by drop casting 50 μL of BSA solution (500 μM) and incubated for 1 hour at room temperature. Electrochemical measurement was conducted after drop casting each concentration of sPDL-1 protein, which was incubated for 45 min at room temperature. Mouse serum was used in the study as it closely replicates the complex biological environment of human blood. This choice allows testing the aptasensor's potential in conditions similar to human physiology. [3,4] For the mouse serum study, different concentrations of sPDL-1 were spiked into mice serum and diluted 10 times with PBS."For the mouse serum study, different concentrations of sPDL-1 were spiked into mice serum and diluted 10 times with PBS. For control samples, 50 µL of non-target proteins such as PD-1 and insulin and biomolecules like glucose and glycine were used. The differential pulse voltammetry (DPV) responses of these control samples were recorded to validate the specificity of the aptasensor for sPD-L1 detection."
5. **Material characterizations:** The surface morphology of the aptasensor was investigated using the scanning electron microscope Zeiss Ultra Plus without a gold coating and a transmission electron microscope JEOL 2100F with a field emission gun of 200 kV. Energy-dispersive X-ray spectroscopy (EDX) was conducted using transmission electron microscopy (TEM), and elemental mapping was acquired over the area of interest. TEM samples prepared by scratching off the electrode surface and deposited on a 200-mesh holey carbon-supported copper grid. The samples ranged from 10 pM, 100 pM, and 100 nM sPD-L1 concentrations. Surface elemental and chemical state analysis was performed using the MAGCISTM X-ray photoelectron spectrometer (Thermo Scientific, MA, USA). Raman spectroscopy was carried out by a

Renishaw inVia™ Raman microscope at 785 nm laser and a 50× objective. The laser power at the sample was 24-240 mW with a laser spot size of *ca*. 1.28 µm. The acquisition time for each spectrum was 10 sec. At least 3 spectra were collected at the different locations on the working electrode. A commercial s-SNOM/nano-FTIR instrument (neaSNOM from neaspec GmbH, Munich) and standard AFM tips (ARROW-NCPT, Nanoworld) with an apex radius of *ca*. 20 nm were used as near-field probes. The spatial distribution of the Au NIs in the sample was investigated using a field-emission scanning electron microscope Zeiss Ultra Plus (operating at 3 kV) without coating.

6. **Electrochemical characterizations:** To validate the stepwise modification of Au NIs/SPCEs, CV and DPV were performed in 5 mM ferricyanide prepared in 0.1 M PBS solution (pH=7.2). In both techniques, the working electrode surface was scanned by restricting the potential window between –0.2 to 0.6 V *vs* Ag/AgCl at a scan rate of 50 mV s^{-1}. the optimized DPV parameters that resulted in a better electrochemical response was the amplitude of 0.05 V, modulation time of 0.05 sec, and interval time of 0.1 sec. All electrochemical measurements were conducted at room temperature.

7. **Statistical analysis:** All data presented here are plotted as mean ± standard deviation with N = 3 for intraassay variation. using the equation LOD=3**Sb***S*, where *Sb* is the standard deviation of the blank and *S* is the calibration curve slope.[5]

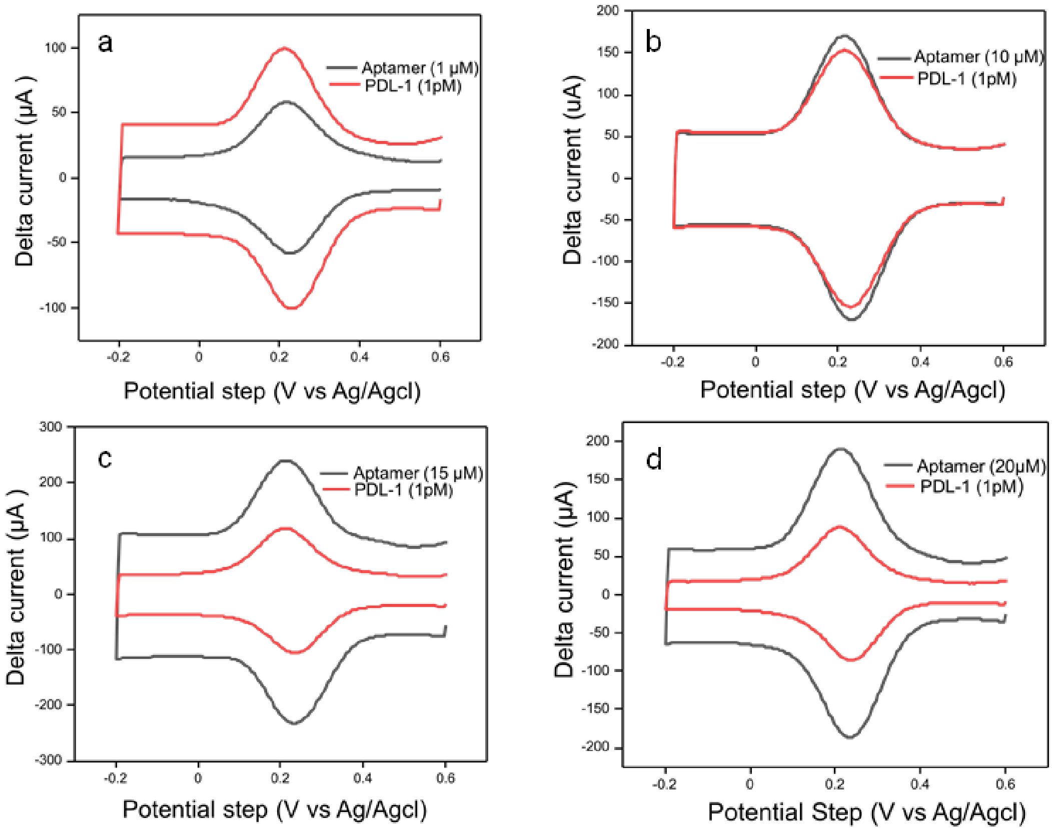

Figure S1. DPV measured in 5 mM ferricyanide solution for different concentrations of binding aptamer: (a) 1 μM, (b) 10 μM, (c) 15 μM, (d) 20 μM immobilised on Au NIs/SPCE.

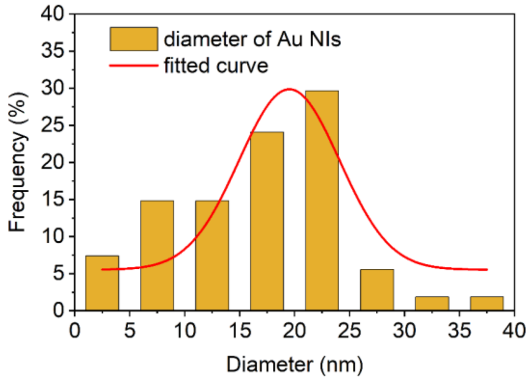

Figure S2. Size distribution of Au NIs on SPCE substrates. Statistical analysis based on TEM image in Figure 3a. The average diameter of the Au NIs centres around 19 nm.

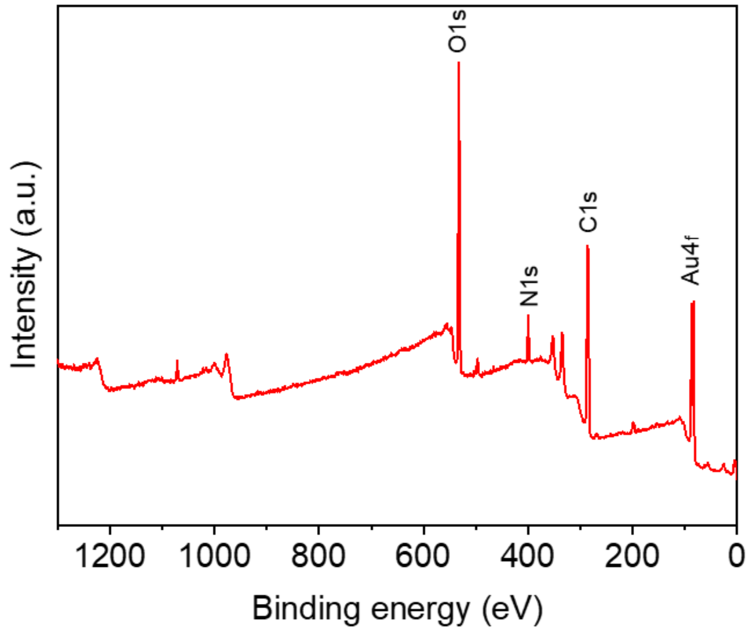

Figure S3. The XPS survey spectrum of aptasensor after exposure to 100 nM sPD-L1 protein.

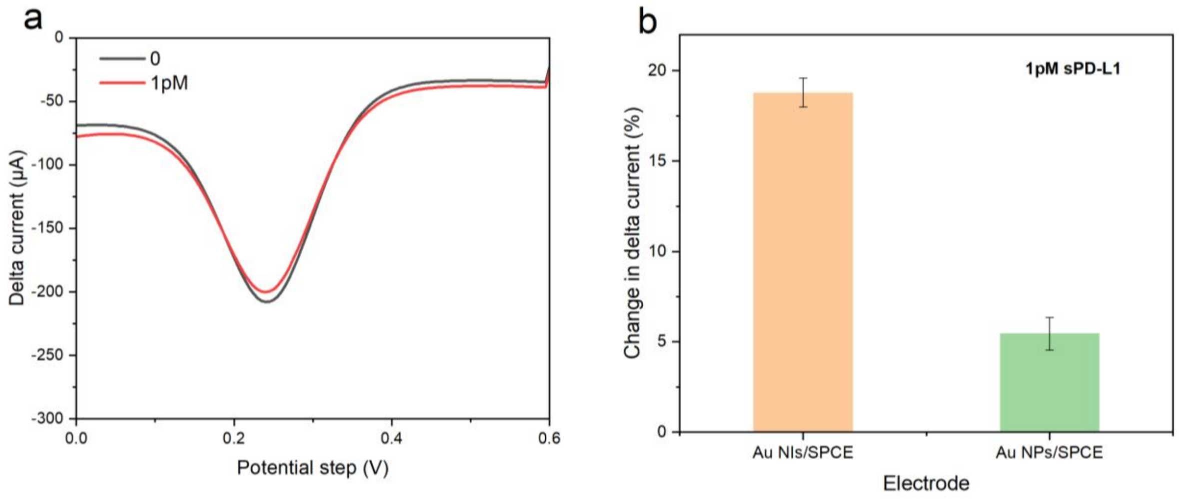

Figure S4. (a) Electrochemical sensing of Au NPs/SPCE, functionalized with DNA aptamers, to detect 1pM sPD-L1 in a 5 mM ferricyanide solution. (b) Comparisonof sensing responses for 1pM sPD-L1 via differential pulse voltammetry (DPV) on Au NIs/SPCE and Au NPs/SPCE.

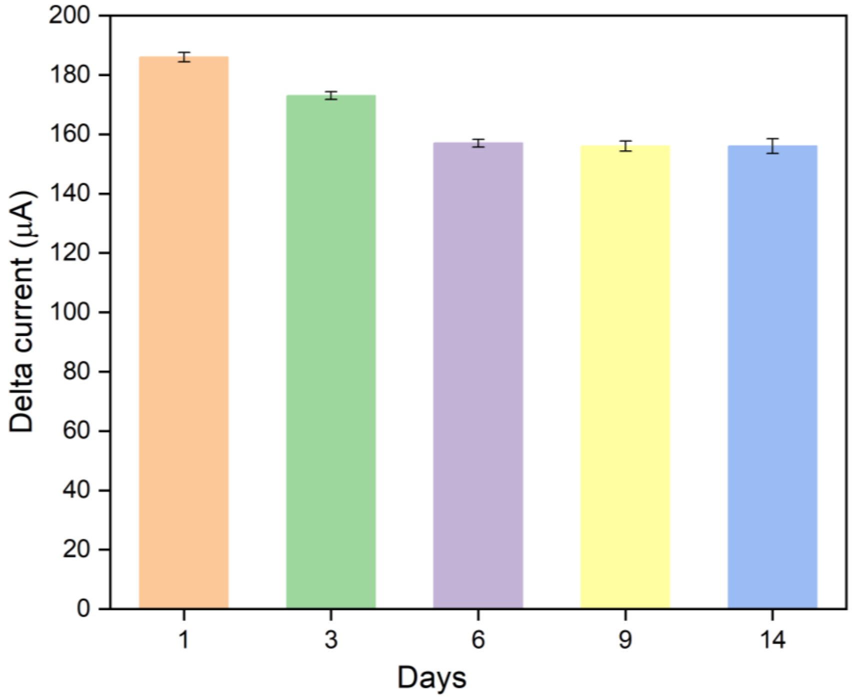

Figure S5. Storage stability of the electrochemical aptasensor for sPD-L1 detection. The bar chart illustrates the aptasensor's performance after 1, 3, 6, 9, and 14 days of storage. Each bar represents the average signal response at these intervals, demonstrating the sensor's consistent sensitivity and reliability over time.

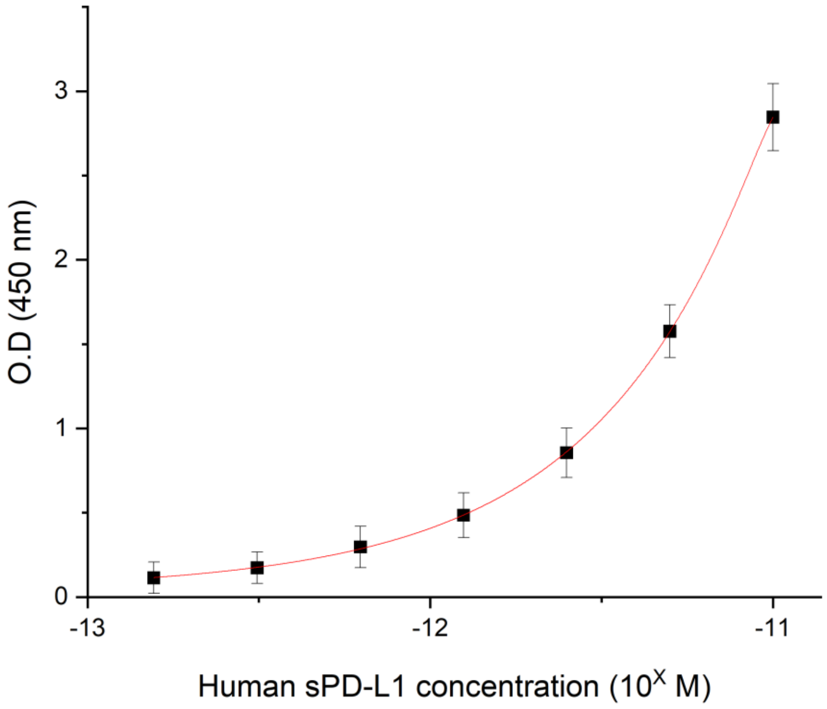

Figure S6. Detection of soluble PD-L1 (sPD-L1) using ELISA. The results show the sPD-L1 levels in the sample, calibrated against a standard curve with known concentrations of recombinant sPD-L1.

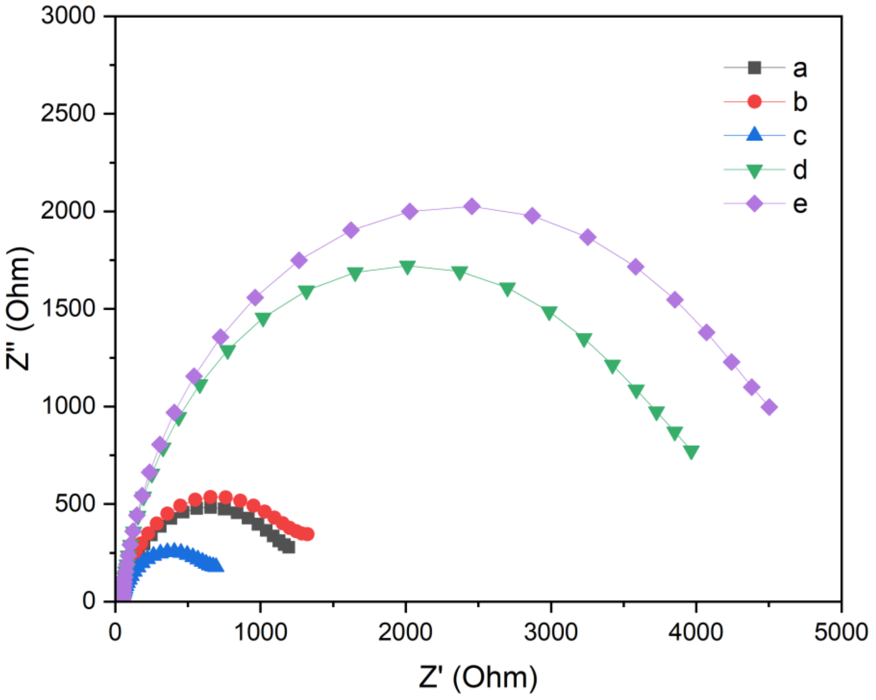

“

Figure S7. Nyquist plots obtained by electrochemical impedance spectroscopy of aptasensor in 0.1M PBS solution containing 5mM ferricyanide: (a) bare Au NIs/SPCE, and Au NIs/SPCE (b) functionalized with aptamer, (c) treated with MCH, (d) treated with BSA, and (e) upon 100 nM sPD-L1 exposure.

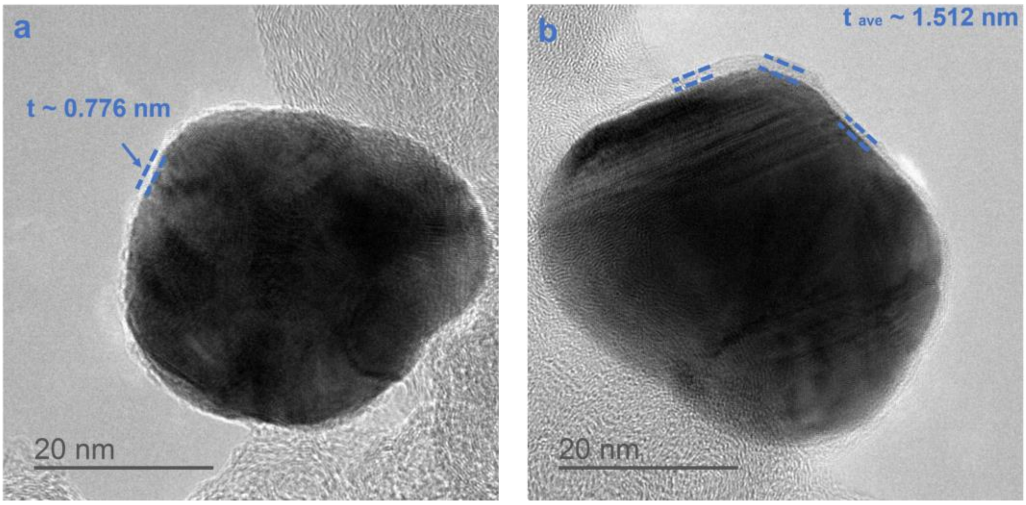

Figure S8. TEM images of functionalized Au NIs upon exposure to (a) 100 pM and (b) 100 nM sPD-L1 solutions. The self-assembled layer's thickness around the Au NIs increases with the sPD-L1 solution concentration. This provides evidence of the increase biomarker interaction demonstrating linking to the observed electrochemical response.

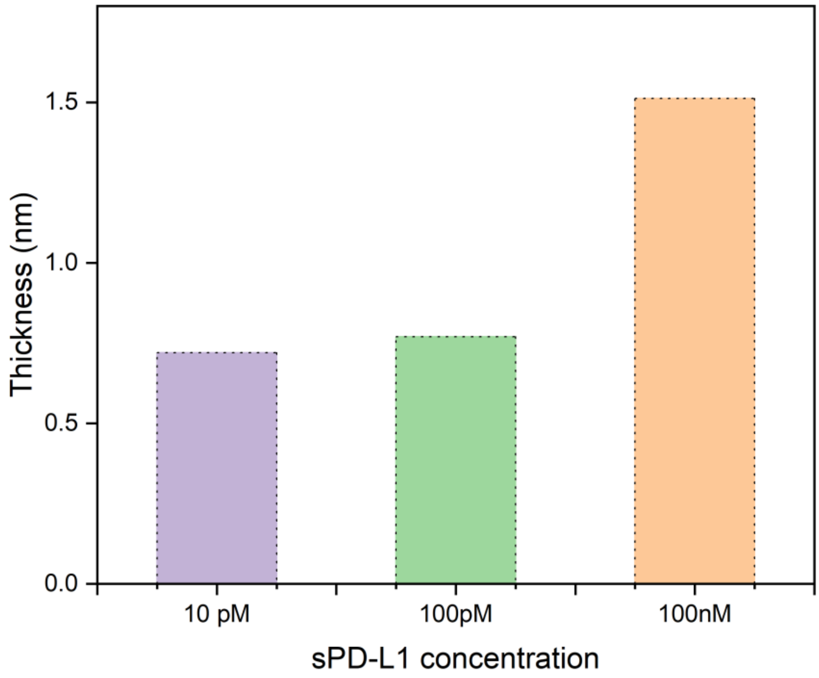

Figure S9. The bar chart represents the thickness of Au NIs measured at three different sPD-L1 concentrations: 10 pM, 100 pM, and 100 nM. The observed thicknesses were 0.72 nm, 0.776 nm, and 1.514 nm, respectively

References

[1] M. G. Dastidar, K. Murugappan, A. M. Damry, D. R. Nisbet, C. J. Nolan, A. Tricoli, Advanced Functional Materials 2022, 32, 2105433.

[2] Jirasirichote, Apapond, et al. "Voltammetric detection of carbofuran determination using screen-printed carbon electrodes modified with gold nanoparticles and graphene oxide." *Talanta* 175 (2017): 331-337.

[3] Wang, Zhenbo, et al. "A novel copper ion enhanced electrochemical DNA biosensor for the determination of epinephrine." *Talanta* 276 (2024): 126274.

[4] Yahia, Yahia A., et al. "Electrochemical Biosensors for Interleukins: Electrode Materials." *Journal* of The Electrochemical Society 170.6 (2023): 067501.

[5] V. Thomsen, D. Schatzlein, D. Mercuro, Spectroscopy (Santa Monica) 2003, 18, 112.
